# Simultaneous Determination of Miconazole Nitrate and Metronidazole in Different Pharmaceutical Dosage Forms by Gas Chromatography and Flame Ionization Detector (GC-FID)

**Published:** 2010-03

**Authors:** Safwan Ashour, Nuha Kattan

**Affiliations:** *Bioanalytical Laboratory, Department of Chemistry, Faculty of Science, University of Aleppo, Aleppo, Syria*

**Keywords:** simultaneous determination, miconazole nitrate, metronidazole, tablets, ovules, gas chromatography

## Abstract

A simple, rapid and precise gas chromatographic method has been developed for the simultaneous determination of miconazole nitrate (MIZ) and metronidazole (MNZ) in tablets and ovules, using a capillary column AE.SE-54 (15 m × 0.53 mm, i.d.) and nitrogen as a carrier gas at a flow rate of 9 mL min^−1^. The oven temperature was programmed at 140°C for 3 min, with a rise of 40°C min^−1^ up to 180°C (held for 2 min) and then increased to a final temperature of 250°C. The injector and detector port temperatures were maintained at 260°C. Detection was carried out using flame ionization detector. Results of assay and recovery studies were statistically evaluated for its accuracy and precision. The retention times were about 3.50 and 12.90 min for MNZ and MIZ, respectively. Linearity ranges were 50.0–6030.0 and 62.5–2000.0 μg mL^−1^ for MNZ and MIZ, with limit of detection values of 2.5 and 3.1 μg mL^−1^, respectively. Correlation coefficients (R^2^) of the regression equations were greater than 0.999 in all cases. No interference from any components of pharmaceutical dosage forms or degradation products was observed. According to the validation results, the proposed method was found to be specific, accurate, precise and could be applied to the simultaneous quantitative analysis of MIZ and MNZ in tablets and ovules.

## INTRODUCTION

Imidazoles are five membered ring structures containing two nitrogen atoms with a complex side chain attached to one of the nitrogen atoms. Imidazoles in current clinical use are clotrimazole, miconazole, econazole and ketoconazole (1, 2). Miconazole nitrate (MIZ), 1-[2,4-dichloro-(b-(2,4-dichlorobenzyloxy) phenethyl] imidazole, possesses a wide antifungal spectrum. It is administered by the troche dosage form or by the intravenous infusion in the treatment of severe systemic fungal infections. It is also applied as a 2.0% cream or powder in infections of nails and skin (3, 4). Metronidazole (MNZ), 2-(2-methyl-nitroimidazol-1-yl) ethanol, is a substance that has a wide range of uses due to its activity against protozoa and anaerobic bacteria (5). Literature survey reveals that both MIZ and MNZ are official in U.S.P. (6) and B.P. (7). Several methods are available for the determination of the latter compounds by high-performance liquid chromatography (HPLC) in different pharmaceutical preparations, either alone (8–13), in combinations of MIZ and MNZ (14, 15) or with other active ingredients (16–23). Various Spectrophotometric methods have been reported for the determination of MIZ (4, 20, 24–27) and MNZ (3, 28–35) from its individual and combined formulations with other active ingredients. Derivative spectrophotometric methods have been reported for the simultaneous determination of MIZ and MNZ in combined dosage forms (14, 36). HPTLC (37, 38), quantitative NMR (40), chemometric (41) and titrimetric (42) methods have been described for the determination of MIZ and MNZ from its individual and combined formulations with other active ingredients. MNZ has been determined by chemiluminescence (43), biamperometry (5) and electroanalysis (voltammetry and polarography) (44–49) in different pharmaceutical dosage forms. MIZ was determined in its oral gel formulation by GC on a column (11 m × 0.22 mm) of CP Sil 5CB (0.12 mm) at 270°C with N_2_ as carrier gas (1 mL min^−1^) and N-P detection (50).

However instrumental facilities of HPTLC, NMR, chemometry, chemiluminescence, biamperometry, polarography and voltammetry being rare as compared to GC and HPLC and there is no method for the simultaneous determination of MIZ and MNZ by GC.

So a new method for the simultaneous determination of MIZ and MNZ from pharmaceutical preparations containing these combinations by GC technique is developed.

## EXPERIMENTAL

### Apparatus and conditions

A 7900 gas chromatograph (GC) equipped with a split/splitless injector and a flame ionization detector (FID) from Techcomp Technologies Inc. (China) was used in this study. The separation was carried out on a AE.SE-54 capillary column (15 m × 0.53 mm i.d., 1 μm film thickness) from Analytical Technology (China). A volume of 5 μL sample was injected, in the detector at 260°C, in the mode at a split ratio of 1:10. GC oven temperature was initially maintained at 140°C for 3min, then programmed to 180°C at a rate 40°C min^−1^ and maintained for 2 min, finally it was raised with a rate of 20°C min^−1^ to a final temperature of 250°C with no final hold. Nitrogen (ultrapure) was obtained from G 1010E nitrogen generator (domnick hunter, England) and used as carrier gas at a constant flow rate of 9.0 mL min^−1^. The detector temperature was set at 260°C. Control valve SGK-2LB (China) for providing the switch and adjustment of air to FID and 5μL gas-tight syringe (SGE Analytical science, Australia) were used.

### Solvents

Analytical grade methanol was purchased from LAB-SCAN (Irland) and used to prepare all solutions.

### Materials

Miconazole nitrate (479.1 g mol^−1^), was supplied by Jiangus NHWA Pharmaceutical Group Co. (China), and its purity was found to be 99.50%. Metronidazole (171.2 g mol^−1^), was supplied by Luotian Hongyuan Biochemical Co. (China), and its purity was found to be 99.42%.

Micoplus ovules supplied by K&C Pharma (Aleppo, Syria), Gyno-D ovules supplied by Sandy pharmaceuticals, and Micozole ovules supplied by BPI (Aleppo, Syria), each ovule was labeled to contains 150mg miconazole nitrate and 500mg metronidazole. Flagyras fort tablets supplied by Razi labs. (Aleppo, Syria), and flagyl tablets supplied by Oubari Pharma (Aleppo, Syria), each tablet was labeled to contains metronidazole 500mg. Micotral ovules supplied by Future Pharmaceuticals (Aleppo, Syria) each ovule was labeled to contains miconazole nitrate 400mg.

### Standard Solutions

A combined standard stock solution of miconazole nitrate (4 mg mL^−1^) and metronidazole (8 mg mL^−1^) were prepared in methanol and stored at 2–8°C. The solution was stable for 2 days at least. Working standard solutions were prepared daily by the appropriate dilution of the stock solution with the same solvent.

### Calibration Curve

A series of working standard drug solutions equivalent to 50.0–6030.0 μg mL^−1^ for MNZ and 62.5–2000.0 μg mL^−1^ for MIZ were prepared by diluting the stock standard solution with the methanol. Standard solutions were found to be stable during the analysis time. To construct the calibration curve five replicates (5 μL) of each standard solution were injected immediately after preparation into the column and the peak area of the chromatograms were measured. Then, the mean peak area was plotted against the corresponding concentration of MNZ and MIZ to obtain the calibration graph (Table 1).

### Assay for ovule formulations

Twenty ovules containing miconazole nitrate and metronidazole were accurately weighed and cut. Five accurately weighed quantities of the cutting ovules equivalent to one ovule were transferred into 50 mL separated beakers. A 25 mL of methanol was then added to each beaker and the mixture was mixed on a hot plate stirrer at 60°C for 15min. After this period, the solution was cooled and filtered using filter paper (Whatman 4, England). Then, the volume of each filtrate was adjusted to 100 mL with methanol. Finally, 5 μL of each diluted sample was injected into the column. Peak area of MNZ and MIZ were then measured for the determination. MNZ and MIZ concentrations in the samples were then calculated using peak data and standard curves.

### Assay for tablet formulations

Twenty tablets containing metronidazole were accurately weighed and finely powdered. Five accurately weighed quantities of the powder equivalent to 500 mg of metronidazole were transferred into 100 mL separated volumetric flasks. A 90 mL methanol was then added to each flask and the mixture was shaken well for 5 min. Then, the volume of each mixture was adjusted to 100 mL with the methanol. The sample solutions were filtered and a suitable concentration was prepared by diluting the filtrates with methanol. Finally, 5 μL of each diluted sample was injected into the column and data were recorded. Metronidazole concentrations in the samples were then calculated using peak data and standard curves.

## RESULTS AND DISCUSSION

### Chromatography

**Figure 1:**
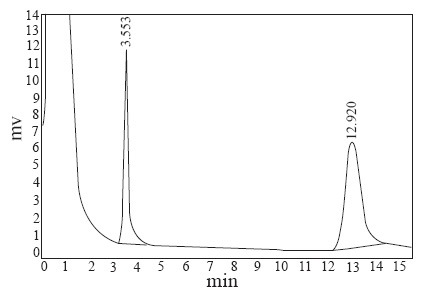
Typical GC-FID chromatogram of 1500 μg mL^−1^ MNZ and 500 μg mL^−1^ MIZ at flow rate 9 mL min^−1^.

The goal of this study was to develop GC assay for the analysis of MNZ and MIZ drugs in pharmaceutical dosage forms. The chromatographic separations were performed on an AE.SE-54 capillary column which gave a good separation of the drugs. Sharp and symmetrical peaks were achieved by programming the oven temperature at 140°C for 3min, then programmed to 180°C at a rate 40°C min^−1^ and maintained for 2 min, finally was raised with a rate of 20°C min^−1^ to a final temperature of 250°C. The retention times of MNZ and MIZ were about 3.50 and 12.90 min, respectively (Fig. 1).

**Figure 2:**
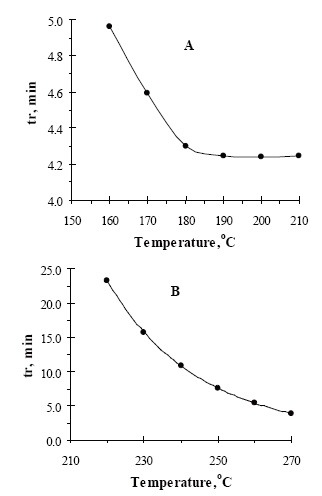
Effect of temperature on the retention time of (A) 500 μg mL^−1^ MNZ and (B) 300 μg mL^−1^ MIZ at flow rate 9 mL min^−1^.

**Figure 3:**
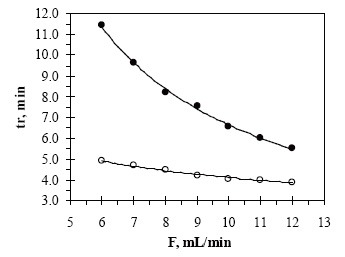
Effect of flow rate on the retention time of (○) MNZ 500 μg mL^−1^ and (•) MIZ 300 μg mL^−1^.

The effect of the oven temperature on the retention time of MNZ and MIZ was investigated and presented in Fig. 2. The effect of carrier gas flow rate in the chromatographic separation of both compounds was also investigated by change the flow rate values of the nitrogen from 6.0 to 12.0 mL min^−1^. For all experimental concentration values, the drugs are separated in order of MNZ and MIZ. A flow rate value of 9.0 mL min^−1^ was chosen for the optimum separation of the compounds, as at this value the analyte peaks were well defined and resolved (Fig. 3).

### Linearity, precision and accuracy

Under the optimal conditions for GC-FID, the calibration curves showed a linear range from 50.0–6030.0 and 62.5–2000.0 μg mL^−1^ for MNZ and MIZ, respectively. The calibration curves could be represented by the linear regression equations:

$$Y_{\text{MNZ}}=0.0595X+2.586\;\;\;\;\;\;\;(R^{2}=0.9999)$$

$$Y_{\text{MIZ}}=0.0849X-3.3829\;\;\;\;\;\;\;(R^{2}=0.9998)$$

where *Y* = area and *X* = concentration of the drug in μg mL^−1^.

As shown in Table 1, the recovery of MNZ and MIZ ranged from 99.8% to 100.9% and 100.1% to 103.0%, respectively, which indicates the method of GC-FID was suitable for the determination of MNZ and MIZ.

**Table 1:** Accuracy and precision for the determination of miconazole nitrate and metronidazole by gas chromatography

Nominal concentration (μg mL^−1^ )	Assayed concentration (μg mL^−1^ )	RSD%	Recovery%	Er%
	Mean^a^ ± S.D.			

Miconazole nitrate				
62.5	64.4 ± 1.3	2.0	103.0	3.0
125.0	125.9 ± 1.2	0.9	100.7	0.7
500.0	501.7 ± 3.4	0.7	100.3	0.3
1000.0	1002.6 ± 3.2	0.3	100.2	0.3
2000.0	2002.9 ± 2.2	0.1	100.1	0.1
Metronidazole				
50.0	49.9 ± 1.0	2.1	99.8	−0.2
187.0	187.7 ± 2.8	1.5	100.3	0.3
375.0	377.5 ± 4.1	1.1	100.6	0.6
750.0	757.3 ± 6.0	0.8	100.9	0.9
1500.0	1503.2 ± 10.5	0.7	100.2	0.2
3000.0	2997.9 ± 17.9	0.6	99.9	−0.1
4000.0	4009.2 ± 20.0	0.5	100.2	0.2
6000.0	6029.2 ± 24.1	0.4	100.4	0.5

a Mean values represent five determinations.

The precision and accuracy of the method were evaluated by analysis of standard solutions of the mixture in replicates of five. The standard deviation, relative standard deviation, recovery and relative error of different amounts tested were determined from the calibration curve.

As indicated in Table 1, the R.S.D. was not higher than 2.1%, and the highest relative error was 3.0%. The satisfied precision and accuracy indicated the good performance of GC-FID for the quantitative analysis of MNZ and MIZ.

### Limits of detection and quantitation

The minimum level at which the investigated compounds can be reliably detected (limit of detection, LOD) and quantified (limit of quantitation, LOQ) were determined experimentally. LOD was expressed as the concentration of drug that generated a response to three times of the signal to-noise (S/N) ratio, and LOQ was 10 times of the S/N ratio. The LOD of MNZ and MIZ attained as defined by IUPAC (51), LOD_(*k*=3)_=*k*× *S*_a_/*b* (where *b* is the slope of the calibration curve and *S*_a_ is the standard deviation of the intercept), was found to be 2.5 and 3.1 μg mL^−1^, respectively. The LOQ was also attained according to the IUPAC definition, LOQ_(*k*=10)_=*k*× *S*_a_/*b*, and was found to be 8.3 and 10.3 μg mL^−1^, respectively.

### Applications

**Figure 4:**
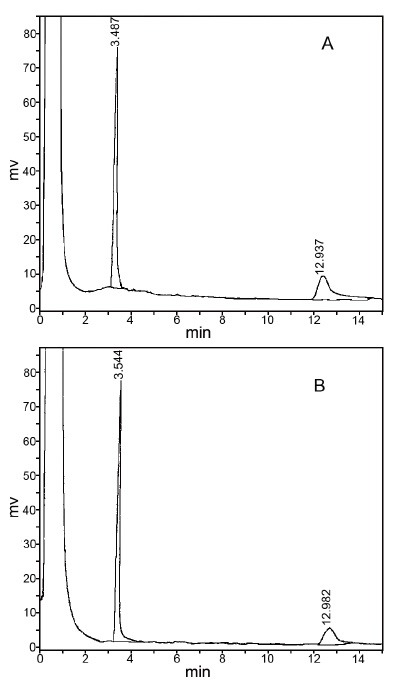
Gas chromatograms from (A) mixture of standard solution of MNZ and MIZ and (B) sample solution prepared from ovules.

The applicability of this GC method to the real samples of MNZ and MIZ was investigated for pharmaceutical preparations. The proposed method was applied for analysis of tablet and ovule samples. Figure 4 shows gas chromatograms from (A) mixture of standard solution and (B) sample solution prepared from ovules. The results obtained by the proposed method are in satisfactory agreement. The analytical results are summarized in Table 2. The low values of R.S.D. indicate that the method is precise and accurate. A blank run of the excepients present per the ovule and tablet of cough syrup, such as Sodium Citrate, ammonium chloride and menthol did not show any interference.

**Table 2:** Analysis of miconazole nitrate and metronidazole in pharmaceutical dosage forms

Preparation	MNZ					MIZ				
	Label claim (mg)	found (mg) a	Conf. lim. b	Recovery %	RSD %	Label claim (mg)	found (mg) a	Conf. lim. b	Recovery %	RSD %

Micoplus	500	500.5	±3.1	100.1	0.56	150	148.8	±1.9	99.2	1.06
Gyno-D	500	495.5	±6.8	99.1	1.11	150	149.4	±2.2	99.6	1.20
Micozole	500	501.5	±5.7	100.3	0.93	150	148.5	±1.5	99.0	0.81
Flagyras fort	500	507.0	±7.6	101.4	1.21	--	--	--	--	--
Flagyl	500	507.0	±5.5	101.4	0.88	--	--	--	--	--
Micotral	--	--	--	--	--	400	401.2	±1.6	100.3	0.33

a Mean values represent five determinations;

b Confidence limits, calculated value for 95% confidence level.

## CONCLUSION

Newly developed GC-FID method was developed for determining metronidazole and miconazole nitrate in pharmaceutical dosage forms. The developed method has proved specific, precise and accurate for assaying the two drugs either individually or in mixtures. The high specificity and resolving power of the developed GC-FID method enabled us to analyze mixtures of the two investigated drugs without the need for pre-separation steps.
